# Temporal Preparation Driven by Rhythms is Resistant to Working Memory Interference

**DOI:** 10.3389/fpsyg.2012.00308

**Published:** 2012-08-28

**Authors:** María Dolores de la Rosa, Daniel Sanabria, Mariagrazia Capizzi, Angel Correa

**Affiliations:** ^1^Departamento de Psicología Experimental, Universidad de GranadaGranada, Spain

**Keywords:** exogenous attention, reaction times, working memory, temporal orienting, bottom-up, stimulus-driven, dual-task

## Abstract

It has been recently shown that temporal orienting demands controlled attention (Capizzi et al., 2012). However, there is current debate on whether temporal preparation guided by regular rhythms also requires the generation of endogenous temporal expectancies or rather involves a mechanism independent of executive control processes. We investigated this issue by using a dual-task paradigm in two different experiments. In Experiment 1, the single-task condition measured reaction time to respond to the onset of an auditory stimulus preceded by either a regular or an irregular auditory rhythm. The dual-task condition additionally included a working memory task, which demanded mental counting and updating. In Experiment 2, the simultaneously WM task was a variant of the Sternberg Task. We hypothesized that, if temporal preparation induced by rhythms did not involve executive processing, it would not be interfered by the simultaneous working memory task. The results showed that participants could anticipate the moment of target onset on the basis of the regular rhythm and, more important, this ability resisted the interference from the double task condition in both experiments. This finding supports that temporal preparation induced by rhythms, in contrast to temporal orienting, does not require resources of executive control.

## Introduction

Temporal preparation consists of the ability to direct attention to a point in time when a relevant event is expected (Coull and Nobre, 1998). The environment provides us with temporal information such as symbolic cues or temporal regularity of certain events (i.e., rhythms), which we can use to build up temporal expectations about stimulus onset and prepare an optimized response at the appropriate moment in time.

Recent studies have investigated the nature of the mechanisms involved in temporal preparation with the aim of dissociating between exogenous and endogenous components. On the one hand, endogenous temporal preparation (“temporal orienting of attention”) depends on the expectations built on predictive temporal information given explicitly by symbolic cues and used to voluntary prepare the response at the expected time. It has previously been related to processes of controlled nature (Coull and Nobre, 1998; Capizzi et al., 2012). On the other hand, it has been shown that temporal preparation can be induced bottom-up, by the temporal regularities provided by regular sequences of stimuli (i.e., rhythms). Regular rhythms would orient our attentional resources in time without the implication of endogenous temporal expectancies, which is reflected by enhanced accuracy and/or faster response to target stimuli (Jones et al., 2002; Sanabria et al., 2011). A relevant issue in research on temporal preparation is to determine the similarities and differences between these two ways to orient attention within the temporal domain.

### Endogenous temporal preparation

Coull and Nobre (1998), based on the Cost and Benefits paradigm (Posner et al., 1980), developed a temporal orienting task adapted to study how attention can be oriented to specific points in time. The procedure consists of a symbolic cue, which explicitly indicates with high probability the time interval or foreperiod (e.g., “*early*” at 400 ms of cue onset, or “*late*” at 1400 ms) at which the target stimuli will occur. For instance, in 75% of trials the temporal cue indicated correctly the moment of target occurrence (i.e., valid trials), whereas in the remainder of trials the target appeared either before or after that cued time (i.e., invalid trial). The results typically show faster reaction times (RTs) in valid relative to invalid trials, mainly at the short foreperiod, which is known as “temporal orienting effect.” This effect is usually reduced or absent at the long foreperiod (see Correa et al., 2004).

Previous research has supported the involvement of controlled processes in temporal orienting. Capizzi et al. (2012) showed that the temporal orienting effect diminished significantly in demanding dual-task conditions. Nevertheless, sequential effects (i.e., faster RTs when the previous interval had either the same or shorter duration than the current interval) did not show any modulation by performing a simultaneous working memory task. It was concluded that temporal orienting involved controlled processing, which was affected by competition for executive resources demanded by the concurrent task. Sequential effects, associated to automatic processing (Los, 1996; Los and Van den Heuvel, 2001; Vallesi and Shallice, 2007; Vallesi et al., 2007), resisted the dual-task interference.

### Exogenous temporal preparation

Temporal preparation can also be induced by temporal regularities of certain events. It has been shown that the presentation of regular sequences of auditory stimulus (i.e., rhythms) enhanced the performance in a pitch discrimination task when the target tone appeared at a time point corresponding to the temporal pattern of the sequence (Jones et al., 2002; see also Lange, 2010). Moreover, cuing time by means of rhythms speeded up responses to a relevant stimulus when it appeared at the moment in time matching the rhythm’s pace (Sanabria et al., 2011). Jones and colleagues have suggested that rhythms induce automatic temporal preparation, since regular repetitions of tone onsets would synchronize the internal attending activity producing an improved response when target stimulus onset continues the rhythmic pattern (Barnes and Jones, 2000).

Rohenkohl et al. (2011) provided further evidence supporting the involvement of exogenous processes in temporal preparation guided by rhythms. They compared temporal preparation guided by rhythms with temporal preparation guided by symbolic cues to dissociate between exogenous and endogenous processes of temporal preparation. Specifically, participants performed a task consisting of a ball moving across the screen until reaching an occluding band. When the ball reappeared, participants were required to discriminate whether the target contained an upright or tilted cross. Participants could predict the moment of target occurrence by means of either the rhythm (i.e., the ball moved following a constant speed, regular rhythmic pace) or the meaning of the symbolic cue (i.e., the color of the ball predicted the duration of the occlusion). At the beginning of the task, participants were instructed to attend to either the rhythm or the symbolic cue to predict the target onset. Results showed that temporal regularity of rhythms enhanced responses to the target regardless of the instructions received by participants (“attend to color” or “attend to speed”). However, the effect of symbolic cues depended on the instruction to attend to color, that is, symbolic cuing was only effective in the “attend to color” but not in the “attend to speed” condition. Therefore, these findings suggested dissociation between temporal preparation driven by rhythms and temporal preparation guided by symbolic cues.

A recent neuropsychological study (Triviño et al., 2011) has shown that patients with right frontal damage could orient attention in time by means of regular rhythms, whereas deficit was observed when symbolic cues were presented. Triviño et al.’s (2011) findings further suggest that temporal preparation guided by rhythms does not depend on the endogenous building up of temporal expectancies. Thus, temporal preparation induced by rhythms would involve a more exogenous bottom-up process, such that it would not depend on the functioning of right prefrontal structures related to attentional control.

In contrast, an event-related potentials (ERPs) study (Schwartze et al., 2011) has questioned the sole involvement of exogenous bottom-up processes in temporal preparation guided by rhythms. Schwartze et al. (2011) used an auditory oddball paradigm to investigate whether regularity of rhythms influenced automatic processing (as indexed by the mismatch negativity – MMN – potential) or “attention-dependent” processing (as indexed by the P3b potential), in two sessions, “pre-attentive” and “attentive.” In both sessions, the auditory sequence could be formed by either a regular or an irregular rhythm (i.e., isochronous vs. random temporal structure). In the pre-attentive session, participants had to watch a video clip while listening to an auditory rhythm that should be ignored. In the attentive session, participants should concentrate on the rhythm and count the deviant tones in each auditory sequence. The results showed that regular rhythms modulated the attention-related potential (P3b) in the attentive session, while in the pre-attentive session, the automatic processing potential (MMN) was not influenced by the rhythm. Schwartze et al. (2011) concluded that synchronization of attention by rhythms required the involvement of top-down mechanisms, such that the influence of temporal regularity was dependent on top-down attentional processing rather than on bottom-up automatic processing. This result differed from previous research suggesting that temporal preparation driven by rhythms involves exogenous bottom-up processing, since it was not necessary to attend to rhythms to orient attention in time (Rohenkohl et al., 2011; Sanabria et al., 2011, Experiment 3).

To summarize, while there is agreement on the controlled nature of the endogenous temporal preparation driven by symbolic cues, it remains currently unclear whether exogenous temporal preparation driven by regular rhythms involves only bottom-up mechanisms or it requires the development of endogenous temporal expectancies. In order to clarify this issue, in the present study, we used a dual-task paradigm to compare the effects of temporal preparation guided by rhythms between a single-task condition and a dual-task condition.

A dual-task paradigm requires performing two tasks (primary and secondary task) simultaneously. In our study, the single-task condition consisted of a temporal preparation task, in which the time of target onset was cued by means of auditory rhythms similar to Lange (2010) who found faster RTs when the target was preceded by a regular rather than by an irregular sequence of six tones. In Lange’s study both sequences of tones had the same duration. Equating duration of both sequences was crucial for the dual-task condition of our study, where, in addition to performing a RT task, participants had to perform simultaneously a WM task. Once the memory retention interval was the same for both rhythm conditions, we could measure the effect of rhythm by comparing the two conditions that are balanced in terms of memory demands. In Experiment 1, the WM task required to count and remember how many times a colored fixation point (from three different colors, changing on a trial-by-trial basis) was presented along a block of trials. In Experiment 2, we aimed to replicate Experiment 1 by using a WM task based on Sternberg’s memory scanning paradigm, in which participants had to remember a new a sequence of six letters in every trial. If performance on the primary task was affected by the secondary task, it could be assumed that both tasks competed for common limited endogenous resources (cf. Posner and Snyder, 1975; Logan, 1978). If temporal preparation driven by rhythms was independent of resources of executive control, its effect on RT would not be affected by performing concurrently the WM task.

## Experiment 1

### Method

#### Participants

Thirty-three undergraduate students (31 females; age range: 19–43 years old; mean age: 22.66 years old) from the Faculty of Psychology of the Universidad de Granada took part in Experiment 1 in exchange of course credits. Participants were randomly assigned to two groups according to task conditions, 16 participants completed the single-task condition and 17 the dual-task condition. In the last condition, one participant was eliminated due to poor performance (22% of correct responses) in the working memory task.

#### Apparatus and stimuli

**Figure 1:**
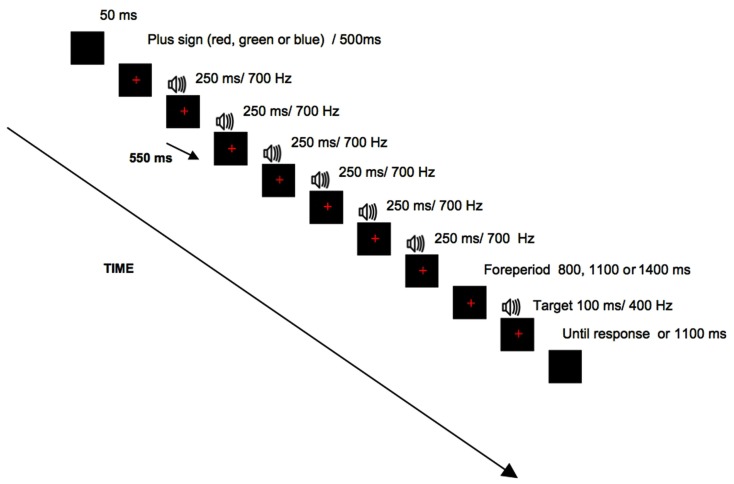
**Schematic representation of events in a trial in the regular rhythm**. In the irregular rhythm the duration of each interval varied and could be either 150, 350, 550, 750, or 950 ms.

Experiment 1 was run on an Intel Core 2 Duo connected to a 17″ LCD monitor. The E-prime software (Schneider et al., 2002) was used for stimulus presentation and to record participants’ responses. The viewing distance was approximately 60 cm. Both single-task and dual-task conditions shared the same auditory and visual stimuli appearing in the center of the screen. In both conditions, the temporal preparation task consisted of a sequence of six tones with duration of 250 ms each and a frequency of 700 Hz. This sequence could be temporally regular or irregular. In the regular sequence, the interval between tones was 550 ms. In the irregular sequence the duration of each interval could be either 150, 350, 550, 750, or 950 ms. The order of these five intervals was randomized across trials. Both sequences included the same number of tones (six tones) and had identical duration, therefore, the only difference concerned temporal regularity or irregularity. The target tone was a 100-ms sound of 400 Hz (Figure 1).

At the beginning of the trial, a plus sign (1.5° × 1.5°) appeared either in red, blue, or green, chosen at random for each trial with the same probability of appearance. All visual stimuli were presented on a black background in the center of the screen.

#### Procedure and task

Both verbal and written instructions were given to participants, who had to press the b key as fast as possible when the target tone was presented. Moreover, they were informed that, before the target, a sequence of sounds forming a rhythm would be presented, which was irrelevant for the task and should therefore be ignored.

Each task condition consisted of one practice block and eight experimental blocks composed of 16 trials each. In both task conditions, each trial began with the presentation of a black screen for 50 ms. Next, a plus sign filled with one of the three colors (red, blue, and green) was randomly generated and remained present during the trial. Then, 500 ms after the plus sign’s appearance, a regular or irregular rhythm was presented at random. The rhythm was followed by the target tone that appeared after a foreperiod of variable duration (800, 1100, and 1400 ms) that was generated at random for each trial. Each foreperiod had a different probability of occurrence based on a non-aging distribution. It consisted of increasing the frequency of the shorter foreperiod such that the conditional probability for target appearance remained constant through the trial. The target tone appeared at the 800-ms foreperiod, in 50% of the trials, at the 1100 ms foreperiod, in 25% of the trials and at the 1400-ms foreperiod, in 12.5% of the trials. In the remaining 12.5% of trials, the target tone was not presented (catch trials). Participants had a maximum of 1150 ms to respond and in case of responding before target onset, a message provided visual feedback on anticipatory error.

In the dual-task condition, the procedure was similar to the single-task condition, except for that participants should perform simultaneously a WM task. The WM task consisted of remembering how many times each color appeared during a block of trials. At the end of each block, participants should type how many times a certain color (e.g., “*green*”) had been presented. Each color was selected at random and with the same probability for the memory test. When participants responded, a message provided feedback about memory accuracy. The word “correct” or “incorrect” filled in green and red color respectively, was presented for 1500 ms.

#### Design and data analysis

The Experiment 1 constituted a 2 × 3 × 2 design with independent variables of Rhythm (regular and irregular) and Foreperiod (800, 1100, and 1400 ms) as within participants factor and Task (single and double) as a between participants factor.

Practice trials, premature responses (i.e., participants responded before the target appeared), trials with RT below 150 ms and above 1200 ms (0.14% of trials) were eliminated from the analyses. Participants’ mean RTs were analyzed by a repeated-measures analysis of variance (ANOVA).

#### Results

In the WM task, participants’ mean accuracy to the color memory test was 89% (7% SD). In the dual-task condition, RT was analyzed only from correct responses in the memory test, in order to assure that participants were actually engaged in the dual-task condition. The RT from responses in the memory test were not included in analyses.

Mean RTs included in the analyses are detailed for each experimental condition in Table 1.

**Table 1:** **Mean RTs for each Foreperiod (800, 1100, 1400 ms), Rhythm (regular, irregular), and Task condition (single-task, dual-task)**.

	Regular rhythm			Irregular rhythm		
	800	1100	1400	800	1100	1400
Single-task	332 (15)	324 (15)	354 (15)	371 (14)	348 (16)	355 (15)
Dual-task	422 (25)	402 (22)	416 (22)	450 (22)	431 (25)	416 (24)

*Values in parentheses are standard error of the mean*.

**Figure 2:**
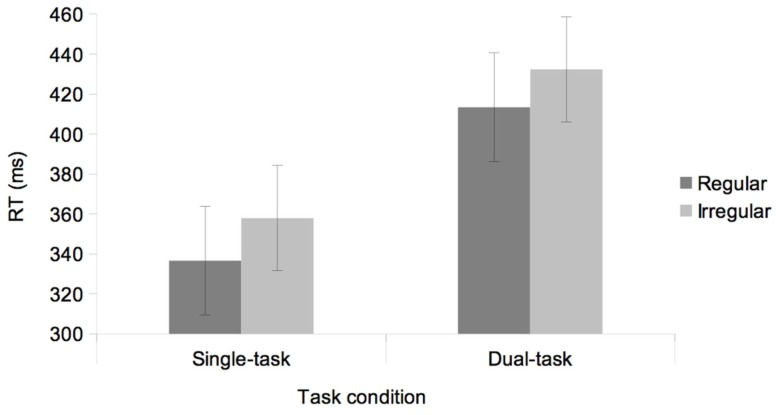
**Mean RTs as a function of Rhythm (regular, irregular) and Task condition (single, dual)**. Error bars represent the standard error of the mean.

The ANOVA showed a significant main effect of Task, *F*(1, 30) = 7.85, *p* < 0.01, with faster RTs in the single-task condition (347 ms) than in the dual-task condition (422 ms). The main effect of Rhythm was also significant, *F*(1, 30) = 51.50, *p* < 0.001, with faster RTs in the regular rhythm (375 ms) than in the irregular rhythm (395 ms). The most relevant finding was that the effect of rhythm did not rely on task condition, since the interaction between Rhythm and Task was not significant, *F* < 1 (see Figure 2). Specifically, the effect of Rhythm was significant in both the single-task, *F*(1, 30) = 20.88, *p* < 0.001, $\eta_{p}^{2}:\text{ 0}\text{.51,}$ and the dual-task condition, *F*(1, 30) = 22.80, *p* < 0.001, $\eta_{p}^{2}:\text{ 0}\text{.79}$.

The main effect of Foreperiod, *F*(2, 60) = 7.60, *p* < 0.01, showed faster RTs in the 1100-ms interval (376 ms) than in the 800-ms (393 ms) and 1400-ms (385 ms) intervals. Planned comparisons indicated a significant difference between the 800 and 1100 ms intervals, *F*(1, 30) = 33.67, *p* < 0.001, whereas the difference between 1100 and 1400 ms intervals was marginally significant, *F*(1, 30) = 3.61, *p* = 0.06. The difference between the 800 and 1400 ms intervals was not significant, *F*(1, 30) = 2.50, *p* = 0.12.

**Figure 3:**
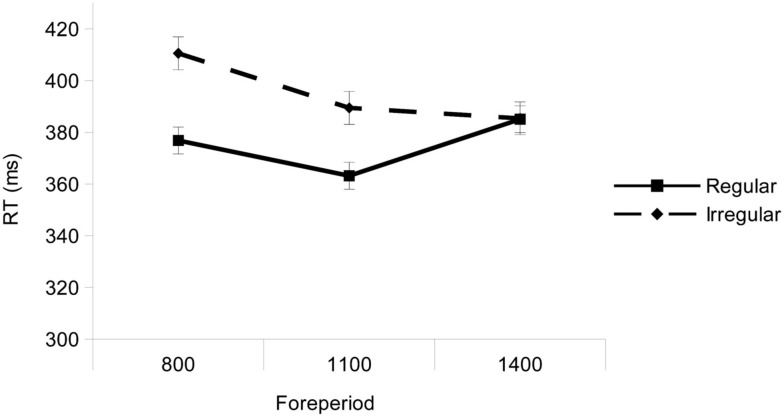
**Mean RTs as a function of the Rhythm (regular, irregular) and Foreperiod (800, 1100, and 1400 ms)**. Error bars represent the standard error of the mean.

The interaction between Rhythm and Foreperiod was significant, *F*(2, 60) = 13.59, *p* < 0.001 (see Figure 3). Planned comparisons between regular and irregular rhythms at each interval, revealed a significant effect of Rhythm in both the 800-ms interval, *F*(1, 30) = 74.40, *p* < 0.001, and the 1100-ms interval *F*(1, 30) = 26.12, *p* < 0.001, but not in the 1400-ms interval, *F* < 1.

Finally, the interaction between Foreperiod and Task also showed a significant result, *F*(2, 60) = 3.59, *p* = 0.03. Further comparisons revealed that RT performance between the two tasks was significant at all foreperiods, *F*(1, 30) = 9.35, *p* < 0.01, *F*(1, 30) = 8.39, *p* < 0.01, and *F*(1, 30) = 5.25, *p* = 0.02, for the 800, 1100, and 1400 ms foreperiods, respectively. Moreover, in the single-task condition, responses were faster in the 1100 interval (335 ms) than both in the 800-ms interval (351 ms), *F*(1, 30) = 13.63, *p* < 0.001, and in the 1400-ms interval (354 ms), *F*(1, 30) = 7.58, *p* < 0.01. The difference between the 800 and 1400 ms intervals was not significant, *F* < 1. In the dual-task condition, planned comparisons showed significant differences between the 800 (435 ms) and 1100 ms (416 ms), *F*(1, 30) = 20.38, *p* < 0.001, and between the 800 and 1400 ms (416 ms) intervals, *F*(1, 30) = 6.69, *p* < 0.01 however, the difference between the 1100 and 1400 ms intervals was not significant, *F* < 1.

### Discussion

Experiment 1 confirmed that participants could use rhythms for temporal preparation even though they simultaneously performed a WM task. The effect of rhythm did not depend on WM load, according to a non-significant interaction between Load and Rhythm. Moreover, the main effect of Task showed faster RT in the single-task condition relative to the dual-task condition showing therefore an effective manipulation of the memory task.

In the Rhythm by Foreperiod interaction found in the Experiment 1, temporal preparation guided by rhythms was selective such that RTs were faster in the regular rhythm than in the irregular rhythm condition in the 1100-ms interval, that matched (two steps of) the regular sequence, and in the 800-ms interval (see next paragraph for Discussion on this finding). However, no rhythm effect was found in the 1400-ms interval. The current data diverged from the findings by Lange (2010), where the interaction between rhythm condition and foreperiod showed that participants’ responses were faster at the 1400-ms interval, probably due to the variable foreperiod effect (i.e., faster RTs at longer intervals) in her experiment. For this reason, we used a non-aging distribution with catch trials where *a priori* probability of occurrence was larger in the shortest foreperiod, thus holding the same conditional probability of target onset throughout the trial that is, this manipulation increased the uncertainty on the moment of target onset, preventing the foreperiod effect (cf. Sanabria et al., 2011).

The rhythm effect was not restricted to the 1100-ms interval, but it was also observed at the 800-ms interval. This result replicates similar findings of previous studies (Griffin et al., 2001; Sanabria et al., 2011), in which a temporal preparation effect was found at the interval shorter than the inter-onset intervals of the sequence. These results have been interpreted as an anticipatory effect (Griffin et al., 2001), that is, an efficient strategy that would consist of preparing for around the shortest foreperiod and then extending preparation to the following foreperiod. However, this result could be interpreted in two different ways: on the one hand, the irregular rhythm would impair temporal preparation at the 800-ms interval where larger RTs were observed. On the other hand, since the mean duration in both sequences was the same, it would improve temporal preparation at the 1100-ms interval that matched the temporal pattern (i.e., two steps of the mean duration), in both sequences. Future research is required to reveal whether the rhythm effect in the irregular sequence was produced by improvement in the temporal preparation at the 1100-ms interval or impairment at the 800-ms interval.

The Foreperiod by Task interaction reached statistical significance, which revealed significant differences in RT between the 1100-ms foreperiod with respect to the other two foreperiods only in single-task condition. In the dual-task condition, participants responded faster in the 1100-ms foreperiod than in the 800-ms foreperiod, but not faster than in the 1400-ms foreperiod. It would appear then that the response enhancement at the foreperiod matching (two steps) of the rhythm was somehow reduced in the dual-task condition. Capizzi et al. (2012) reported an incremented foreperiod effect in the dual-task condition with respect to the single-task condition. Taken together, Capizzi et al.’s (2012) results and the present findings support the notion that the foreperiod effect results from the action of endogenous temporal preparation, since, in contrast to the rhythm effect, it was affected by the concurrent working memory task. In any case, the foreperiod by task interaction was secondary for the main purpose of our study, and at present, data are not conclusive regarding the nature of the mechanisms involved in the foreperiod effect (Los and Van den Heuvel, 2001; Vallesi and Shallice, 2007; Capizzi et al., 2012).

Although the findings in Experiment 1 suggest the involvement of bottom-up processing (i.e., in opposition to top-down executive control processing) in the temporal preparation driven by rhythms, it is possible that our load manipulation was not optimal to produce strong interference. In the current WM task, memory load was not constant along the block, so that at the beginning the number of colors to be remembered was lower than at the end of the block. Therefore, it would allow paying attention to the rhythms providing an optimal attentional preparation in time. Thus, we designed a new task in Experiment 2 in which the demands of the WM task were the same during the whole block. Specifically, we followed a procedure based on the Sternberg’s memory scanning paradigm (Sternberg, 1966). This task consisted of presenting a sequence of six consonant letters (memory list) that participants had to remember during the trial. At the end of the trial, a letter selected at random (probe) was presented and participants had to respond whether that letter was present or absent from the initial memory list. This manipulation involved two task conditions with respect to the memory load: the Low load condition, where the memory list was formed by the same letter, and the High load condition in which the memory list was formed by six different letters. In the case of Experiment 2 the task load conditions were manipulated within participants, thus increasing statistical power to study our main effect to interest.

The RT task consisted of regular and irregular rhythms identical to Experiment 1, but a control condition was further included in which no rhythm was presented (instead, the trial was silent and the only stimulus presented to the participant was the initial fixation point and the last target tone). The idea was to test whether regular and irregular rhythms produced benefits or costs on the temporal preparation based on rhythms.

First, we expected to find that participants could prepare in time by mean of rhythms, showing enhanced RTs in the regular rhythms in comparison to irregular and no rhythm conditions. Moreover, if the secondary Sternberg task did not affect the ability for temporal preparation, this would further suggest that temporal preparation guided by rhythms did not require controlled resources for the generation of endogenous temporal expectancies, specifically meaning that it required other resources than those for WM executive control.

## Experiment 2

### Method

#### Participants

Eleven undergraduate students (10 females; age range: 18–24 years old; mean age: 20.64 years old) participated voluntarily and in exchange of course credits in Experiment 2.

#### Apparatus and stimuli

Experiment 2 comprised the same stimuli as Experiment 1, except for following differences. The fixation point was presented for 500 ms. In addition to the regular and irregular rhythms, a control condition was included in which no sequence of tones was presented. The duration prior to the foreperiod was identical to those of the regular and irregular rhythms (i.e., 2750 ms). The presentation of each condition (regular, irregular, and no rhythm) was equally likely and randomized across trials. The foreperiod presented a constant duration across the trials, 1100 ms long. Given that in Experiment 1 we showed evidence of the selective and enhanced response in the interval that matched the regular sequence (1100 ms), in Experiment 2 we decided to use only one foreperiod for the sake of simplicity.

The stimuli of the memory task consisted of a set of six letters generated at random among the consonants of the alphabet. This set could contain either the same (e.g., “*ssssss*” – Low load condition) or different letters (e.g., “*nspdmc*” – High load condition). Both Task load conditions were presented at random across trials and with the same probability of occurrence.

#### Procedure and task

As in Experiment 1, participants had to respond to the target tone pressing the space and to ignore the rhythms. After their response to the target tone, a letter was presented on the screen. They were instructed to press the “a” key if that letter was included in the set presented at the beginning of the trial or, on the contrary, press the “z” key, if the letter was not present in the previous set.

The task consisted of one practice block and seven experimental blocks composed of 24 trials each. At the beginning of each trial, the fixation point was presented for 500 ms. Then, the set of six digits appeared for 3000 ms preceding the presentation of the sequence of six tones that could be either the regular, irregular, or no rhythm condition. Next, the target tone was presented after the foreperiod of 1100 ms. When participants responded to the target, the letter for the memory task was displayed on the screen. The inter-trial interval was set to 1100 ms.

#### Results

An ANOVA was conducted on participants’ mean RT with the independent variables of Task load (High and Low) and Rhythm (regular, irregular, no rhythm) as within participants factors. Practice trials, premature responses, and trials with RT below 150 and above 1200 (2.66% of trials) were discarded from analyses. Participants’ mean accuracy to the memory test was 89% (6% SD). As in Experiment 1, the analyses only included correct responses in the memory test.

The ANOVA showed a statistically significant main effect of Load, *F*(1, 10) = 11.27, *p* < 0.001, showing slower RTs in the High load condition (389 ms) than the Low load condition (365 ms). The main effect of Rhythm was also significant, *F*(2, 20) = 27.21, *p* < 0.001, indicating faster RTs after the regular rhythm (338 ms), relative to the irregular rhythm (378 ms) and no rhythm conditions (415 ms; see Table 2). Planned comparisons showed that the difference between regular and irregular rhythms was significant, *F*(1, 10) = 17.45, *p* < 0.01. Both the regular vs. no rhythm and irregular vs. no rhythm differences reached the statistical significance, *F*(1, 10) = 82.94, *p* < 0.001 and *F*(1, 10) = 8.29, *p* < 0.02, respectively.

**Table 2:** **Mean RTs for each Load (high, low) and Rhythm (regular, no, irregular)**.

	Regular rhythm	No rhythm	Irregular rhythm
High load condition	346 (29)	422 (28)	397 (22)
Low load condition	329 (21)	407 (26)	397 (24)

*Values in parentheses are standard error of the mean*.

**Figure 4:**
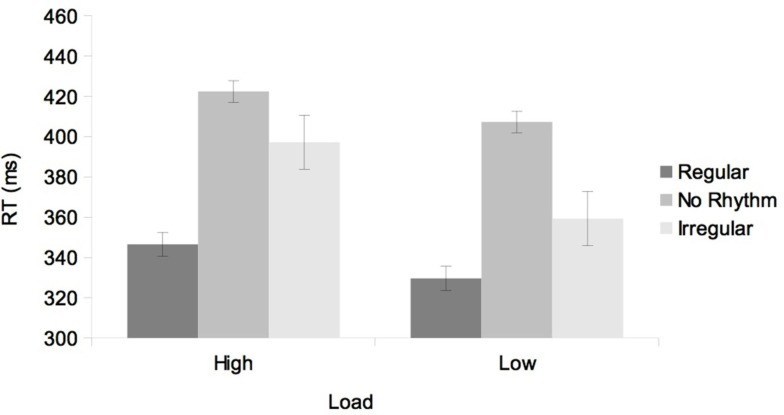
**Mean RTs as a function of Load (high, low) and Rhythm (regular, no rhythm, and irregular) in Experiment 2**. Error bars represent the standard error of the mean.

Most important, the interaction between Load and Rhythm was not statistically significant, *F* < 1 (see Figure 4). In any case, we further analyzed this interaction using planned comparisons. These analyses showed that the regular vs. irregular difference was significant in both Load conditions, *F*(1, 10) = 10.77, *p* < 0.01, $\eta_{p}^{2}\text{ = 0}\text{.52}$ in the high load condition and *F*(1, 10) = 6.33, *p* < 0.03, $\eta_{p}^{2}\text{ = 0}\text{.39}$ in low load condition. The difference between regular and no rhythm was significant in the high load condition, *F*(1, 10) = 28.23, *p* < 0.001, and in the low load condition, *F*(1, 10) = 37.66, *p* < 0.001. Finally, the irregular and no rhythm difference was significant in the low load condition, *F*(1, 10) = 12.96, *p* < 0.01 but in the high load condition it did not reach the statistical significance, *F*(1, 10) = 1.81, *p* = 0.21.

### Discussion

The finding of a main effect of Rhythm confirmed that participants could temporally prepare attention by means of regular rhythms. Such rhythm effect was found of a similar magnitude in both the low and high memory load condition confirming that temporal preparation was preserved in the dual-task condition.

Importantly, contrary to Experiment 1, the memory load in Experiment 2 was manipulated in a trial-by-trial manner. The inclusion of a no rhythm condition revealed that even the presence of an irregular rhythm resulted in a benefit in terms of RT performance. It would appear then that the mere presence of the auditory sequence, either regular or irregular, served as a temporal cue for the upcoming target, compared to a condition in which no stimulation was presented prior to the target onset. Crucially, RTs were significantly faster in the regular than in the irregular rhythm condition in both memory load conditions.

## General Discussion

It is currently unclear whether temporal preparation guided by rhythms involves exogenous bottom-up (e.g., Rohenkohl et al., 2011; Sanabria et al., 2011; Triviño et al., 2011), endogenous top-down mechanisms (e.g., Schwartze et al., 2011) or both. The aim of both experiments was to investigate this question by using the dual-task methodology. Assuming that temporal preparation induced by rhythms did not require controlled processing for the building up of endogenous temporal expectancies, the rhythm effect in a simple RT task would not be affected by interference from the WM task. The results in Experiments 1 and 2 showed that participants could prepare in time by means of regular rhythms, resulting in faster RTs in comparison to the irregular rhythm condition (and no rhythm condition in Experiment 2). More relevant, the rhythm effect was present in the high load and low load conditions of both experiments.

An important question to take into account in both experiments concerns to whether the two concurrent tasks involved similar or different sensory modalities. According to the Multiple Resources model (Wickens, 2008), the maximum interference occurs when the two tasks involve stimulus processing within the same sensory modality, as was the case of Capizzi et al. (2012), where both temporal preparation and WM tasks implied visual processing only. Instead, it could be argued that temporal preparation in Experiment 1 of the present study was achieved because the rhythms and the to-be-remember color stimuli did not share the same modality. One could even argue that this was the case in Experiment 2, since the letters in the memory task were presented visually. However, previous research has shown that visual stimuli are kept in short-term memory into a phonological store and that this information is refreshed by subvocal articulation through a process of rehearsal (see Baddeley, 1992, for a discussion).

A recent fRMI study (Habeck et al., 2012) has investigated, by means of the Delayed-Item-Recognition task, the neural substrates involved in non-verbal and verbal visual stimuli. In order to identify the neural regions involved in the non-verbal visual WM, these authors carried out a task consistent of the presentation of a list with one, two, or three abstract line drawings during 3 s and then, a memory test was presented in which participants had to indicate whether the probe stimulus was previously presented. Similarly, another task was performed to identify the neural regions underlying the verbal visual WM, in which letters were used instead of lines drawings. The results showed that in both tasks, verbal and non-verbal, similar frontoparietal brain regions including Broca’s area (i.e., the left inferior frontal gyrus) were active. Habeck et al. (2012) suggested that this area would be involved in articulatory rehearsal of verbalizable information regardless of sensorial modality of the to-be-remembered stimuli. Crottaz-Herbette et al. (2004) also reported similar frontal activation for auditory and visual verbal (non-spatial) working memory tasks, suggesting a common neural substrate for working memory rehearsal irrespective of the modality of presentation of the WM stimuli.

In light of Habeck et al.’s (2012) and Crottaz-Herbette et al.’s (2004) results we could have expected a similar outcome in the present study whatever the modality of the stimuli in the WM. Interestingly, Crottaz-Herbette et al. (2004) also reported deactivation (with respect to a control non-WM condition) of the superior and middle temporal auditory cortex during the visual WM task and deactivation of the occipital cortex during the auditory WM task (cf. Laurienti et al., 2002). Therefore, one would have expected a reduced effect of the rhythm cue in the double task condition in Experiment 1 and in the high load condition in Experiment 2 with respect to the single-task and low load conditions. In contrast, no effects of the concurrent WM over temporal preparation driven by rhythms was found in either experiment. In sum, it would appear then that our main result could not be accounted for solely by a difference in the sensory modality of the stimuli in the WM with respect to the simple RT task.

Our results suggest that the simple auditory RT task and the WM task, both in Experiments 1 and 2, did not compete for the same processing resources. This confirms our main hypothesis that performance in the concurrent WM task would interfere performance in the simple auditory RT task if temporal preparation driven by rhythms would rely on executive processing, which it did not seem to be the case. Note, though, that a concurrent auditory perceptual task could have reduced our main auditory RT effect (cf. Santangelo et al., 2008). However, this would not contradict our main conclusion that is based on top-down executive (WM) processing effects on temporal preparation driven by rhythms.

We therefore argue that temporal preparation driven by rhythms in our study did not entail the building up of endogenous top-down temporal expectancies, in sharp contrast to temporal preparation driven by symbolic cues (cf. Capizzi et al., 2012). Our results adds to the extant literature showing that temporal preparation guided by rhythms is produced in a bottom-up, involuntary way (Rohenkohl et al., 2011; Sanabria et al., 2011; Experiment 3; Triviño et al., 2011) and that is not prone to interference by endogenous controlled processes involved in WM tasks.

The present results can be interpreted according to the dynamic attention model of Jones and colleagues (Barnes and Jones, 2000; Jones et al., 2002), where attention can be exogenously captured by rhythms and directed to appropriate moments in time. Specifically, this model assumes that the temporal pattern of rhythms produces automatically an attentional synchrony, which would enhance responses to stimuli presented at the optimum point in time.

In contrast, Schwartze et al. (2011) showed that stimulus-driven synchronization of attention would rely on top-down attention mechanisms. It is interesting to note that the pre-attentive condition in the Schwartze et al.’s (2011) study might be analogous to our dual-task condition. In effect, in both experiments participants had to concentrate in a secondary task while simultaneously listening to a rhythm (regular or irregular) that should be ignored, although in our memory dual-task condition participants were asked to respond to the target onset. In the pre-attentive condition of the Schwartze et al.’s (2011) study, the regular rhythm did not influence automatic auditory processing as revealed by the MMN potential. In contrast, the regular rhythm only influenced potentials related to attentional processing (P3b potential), and this modulation was selective to the attentive condition. These findings suggest that rhythmic, stimulus-driven, synchronization was produced by mechanisms dependent on top-down attentional mechanisms. Interestingly, behavioral performance in the attentive condition in Schwartze et al.’s (2011) study was not significantly different when comparing the regular and irregular rhythm conditions. However, in our research, we observed a rhythm RT effect (i.e., faster for the regular rhythm than for the irregular rhythm) under dual-task conditions, showing that participants could prepare in time by means of regular rhythms even though they were instructed to ignore the sequence of sounds and to attend to the WM task. Future ERP research would be interesting to clarify these apparently contradictory results, investigating how regular rhythms modulate neural processing in a dual-task condition similar to that of Experiment 2.

In sum, our study showed that temporal preparation driven by rhythms resisted the WM task interference, since participants could prepare in time while they simultaneously performed a secondary task. Thus, our study supports that temporal preparation induced by rhythms, in contrast to temporal orienting, involves stimulus-driven attentional processing in the sense that it does not require resources of executive control.

## Highlights

(1) We investigated whether temporal preparation induced by rhythms relies on automatic mechanisms by using dual-task methodology. (2) Regular rhythms improved RTs to targets appearing at the moment in time matching the rhythmic pace. (3) This behavioral improvement resisted interference when performing concurrently a working memory task. (4) It is concluded that temporal preparation guided by rhythms involves automatic mechanisms.

## Conflict of Interest Statement

The authors declare that the research was conducted in the absence of any commercial or financial relationships that could be construed as a potential conflict of interest.
